# Protocol for whole-mount immunostaining of brains of the eastern subterranean termite, *Reticulitermes flavipes*

**DOI:** 10.1016/j.xpro.2024.103318

**Published:** 2024-09-14

**Authors:** Austin Merchant, Xuguo Zhou

**Affiliations:** 1 Department of Entomology, Martin-Gatton College of Agriculture, Food and Environment, University of Kentucky, Lexington, KY 40546, USA; 2 Department of Entomology, School of Integrative Biology, College of Liberal Arts & Sciences, University of Illinois Urbana-Champaign, Urbana, IL 61801, USA

**Keywords:** Microscopy, Model Organisms, Neuroscience

## Abstract

Immunostaining is a powerful technique for visualizing tissue morphology and protein expression patterns, but in non-model organisms, it may be impeded by a lack of established protocols. Here, we present a protocol for whole-mount immunostaining of termite brains that we applied to the termite *Reticulitermes flavipes*. We describe steps for brain dissection, fixation, staining, and mounting. This approach generates stained termite brains that can be visualized through confocal microscopy and can potentially be adjusted to suit other insect species.

For complete details on the use and execution of this protocol, please refer to Merchant and Zhou.^1^

## Highlights

• Whole-brain immunostaining protocol for use in *Reticulitermes* termites
• Steps for dissecting, fixing, staining, and mounting termite brains
• Produces immunostained brains that can be visualized via confocal microscopy

## Abstract (editor highlights)

Publisher’s note: Undertaking any experimental protocol requires adherence to local institutional guidelines for laboratory safety and ethics.

## Graphical abstract

**Figure:**
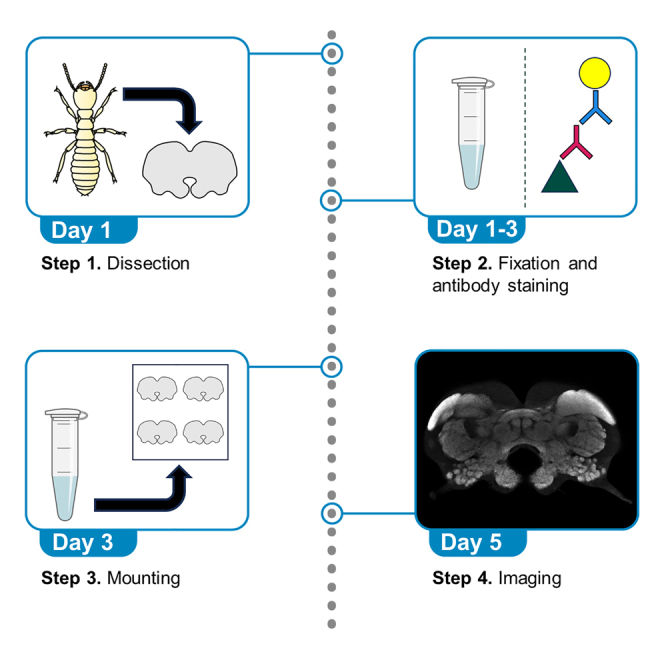

## Before you begin

The brain plays a vital role in controlling insect behavior, yet despite this importance remains poorly understood beyond a basic organizational level in many non-model organisms.^2^ Immunostaining is a commonly applied technique in model insects such as *Drosophila* that can be used to visualize the entire brain as well as specific populations of neurons.^3^^,^^4^^,^^5^ During immunostaining, brain tissue is incubated in a primary antibody that binds to a protein of interest. Tissue is then commonly incubated in a secondary antibody, which binds to the primary antibody and possesses a tag that can be visualized using confocal or epifluorescence microscopy.^6^

Termites, despite their ecological dominance and economic significance,^7^ are one such non-model insect for which, until recently, little information existed regarding the brain. In our study, we imaged the brains of individuals from different castes of one of the most widely distributed termite species in the world, the eastern subterranean termite, *Reticulitermes flavipes*, and compared differences in brain allometry between castes.^1^ To do this, we adapted a whole mount immunostaining protocol from *Drosophila*, which possesses a brain similar in size to that of *R. flavipes*, that uses the commercially available antibody nc82.^3^ nc82 targets the protein Bruchpilot, which is expressed at presynaptic active zones.^8^ In theory, co-staining with other antibodies should also be possible to visualize protein expression patterns within the brain, although we have not directly tested this because for our study we focused on the structure of the brain itself rather than specific proteins. Further adaptation of this protocol to other non-model insects may also be useful as it is relatively inexpensive to perform and is fairly simple except for the dissection and mounting steps, which are likely to require some level of trial and error before producing satisfactory results. Brain tissue is extremely delicate and practical experience is necessary for one to learn precisely where it is located within the target insect’s head and how to remove it with minimal disturbance. The mounting process also requires significant care because it involves physical placement of brains onto a coverslip, where they are at risk of being damaged.

Before carrying out this protocol, please keep the following in mind:

1. All steps should be performed at room temperature (approximately 20°C–25°C) unless noted otherwise.
2. Prepare reagents ahead of time.
3. Equipment listed under “Other” in the key resources table can be substituted with alternatives from other manufacturers, based on price and availability.

### Poly-L-lysine solution preparation

**Timing: 15 min**

Poly-L-lysine (PLL) solution is used to promote adhesion of brains to the coverslip during the mounting step. In order to prepare the solution, a protocol from the FlyLight Project at Janelia Research Campus is used,^9^ described below.

4. Allow poly-L-lysine powder to come to room temperature.
5. Add 2 mL of ultrapure water to the original container, then mix thoroughly until powder dissolves.
6. Transfer contents to a 50 mL conical tube.
7. Add 30 mL of ultrapure water, then mix thoroughly.
8. Add 64 μL of Kodak Photo-Flo 200 Solution, then mix thoroughly.
9. Transfer solution to a glass coverslip jar until it is almost full.
10. Store both the coverslip jar and the conical containing the remainder of the PLL solution at 4°C in total darkness.

***Note:*** Under these conditions, PLL solution can remain usable for up to 12 months.

***Note:*** Coverslips do not need to be prepared in advance and are instead prepared during immunostaining (see step 12 of the “step-by-step method details” below).

***Note:*** PLL solution in the coverslip jar should be reused. If the solution is depleted to the point where it no longer completely immerses a coverslip, add solution to the coverslip jar using the remainder stored in the conical.

## Key resources table

**Table:** 

REAGENT or RESOURCE	SOURCE	IDENTIFIER
**Antibodies**		

Mouse monoclonal anti-Bruchpilot (nc82) (1:50 dilution)	Developmental Studies Hybridoma Bank	Cat#nc82; RRID: AB_2392664
Goat anti-mouse IgG, Alexa Fluor 546 (1:500 dilution)	Thermo Fisher Scientific	Cat#A-11030; RRID: AB_2534089

**Chemicals, peptides, and recombinant proteins**		

Triton X-100	Thermo Fisher Scientific	Cat#85111
1× Phosphate-buffered saline (PBS)	Thermo Fisher Scientific	Cat#10010031
16% Paraformaldehyde (PFA)	Thermo Fisher Scientific	Cat#28908
Normal goat serum (NGS)	Thermo Fisher Scientific	Cat#50197Z
Poly-L-lysine hydrobromide	Sigma-Aldrich	Cat#P1524-25MG
Ultrapure water	Thermo Fisher Scientific	Cat#10977015
Kodak Photo-Flo 200 solution	Kodak	Cat#1464510
Xylene	Thermo Fisher Scientific	Cat#9990501
Ethyl alcohol, 200 proof	Sigma-Aldrich	Cat#EX0276-4
DPX mountant	Thermo Fisher Scientific	Cat#X1525

**Experimental models: Organisms/strains**		

*Reticulitermes flavipes* – field-collected colonies, males & females of castes: worker, soldier, ergatoid, alate, nymph	Daniel Boone National Forest, KY, USA	N/A

**Other**		

Dissection dish	Living Systems Instrumentation	Cat#DD-50-S-BLK
Dissecting forceps	Dumont	Cat#0108-5-PO
Protein LoBind tubes (2 mL)	Eppendorf	Cat#022431102
Conical tubes (50 mL)	Eppendorf	Cat#0030122186
Glass storage bottles (125 mL)	Corning	Cat#CLS1399125
Orbital shaker	Labnique	Cat#MT-201-BD
Microscope slides (75 × 25 mm)	Sigma-Aldrich	Cat#CLS294775X25
Square coverslips (22 × 22 mm)	Sigma-Aldrich	Cat#C9802
Coverslip drying rack	Sigma-Aldrich	Cat#Z688568
Glass coverslip jars (×11)	DWK Life Sciences (Wheaton)	Cat#W900180

## Materials and equipment

**Table:** 10% PBS Triton X-100 (10% PBST)

Reagent	Final concentration	Amount
Triton X-100	10%	5 mL
1× PBS	1×	45 mL
**Total**	**N/A**	**50 mL**

**Storage:** Store at 4°C for up to 3 months inside a 50 mL conical.

**Table:** 2% Paraformaldehyde (2% PFA)

Reagent	Final concentration	Amount
16% Paraformaldehyde	2% (v/v)	10 mL
1× PBS	1×	70 mL
**Total**	**N/A**	**80 mL**

**Storage:** Store at 4°C in total darkness for up to 2 months inside a glass bottle or similar container that can be tightly sealed (an example is listed in the key resources table).

**CRITICAL:** Paraformaldehyde is carcinogenic and should always be handled within a fume hood while wearing gloves.

**Table:** 4% Paraformaldehyde (4% PFA)

Reagent	Final concentration	Amount
16% Paraformaldehyde	4% (v/v)	10 mL
1× PBS	1×	30 mL
**Total**	**N/A**	**40 mL**

**Storage:** Store at 4°C in total darkness for up to 2 months inside a glass bottle or similar container that can be tightly sealed (an example is listed in the key resources table).

**CRITICAL:** Paraformaldehyde is carcinogenic and should always be handled within a fume hood while wearing gloves.

**Table:** Poly-L-lysine solution

Reagent	Final concentration	Amount
Poly-L-lysine hydrobromide	0.078% (w/v)	25 mg
Ultrapure water	99.723% (v/v)	32 mL
Kodak Photo-Flo 200	0.199% (v/v)	64 μL
**Total**	**100%**	**32 mL**

**Storage:** Store at 4°C in total darkness for up to 12 months.

## Step-by-step method details

### Day 1: Brain dissection, tissue fixation, and staining

**Timing: 3 h + 5–10 min per brain**

Brains are dissected, fixed in paraformaldehyde (PFA) to preserve brain tissue, washed in phosphate-buffered saline with Triton X-100 (PBST) to clean the brains of leftover reagents, and incubated in the primary antibody, which binds to the protein of interest (in this case, Bruchpilot). Fixation and staining require a fixed amount of time to complete, while the duration of dissections is reliant on the skill of the dissector and the number of brains dissected. It is recommended to practice dissections beforehand to build experience and gauge the amount of time that they will take.

**Figure 1:**
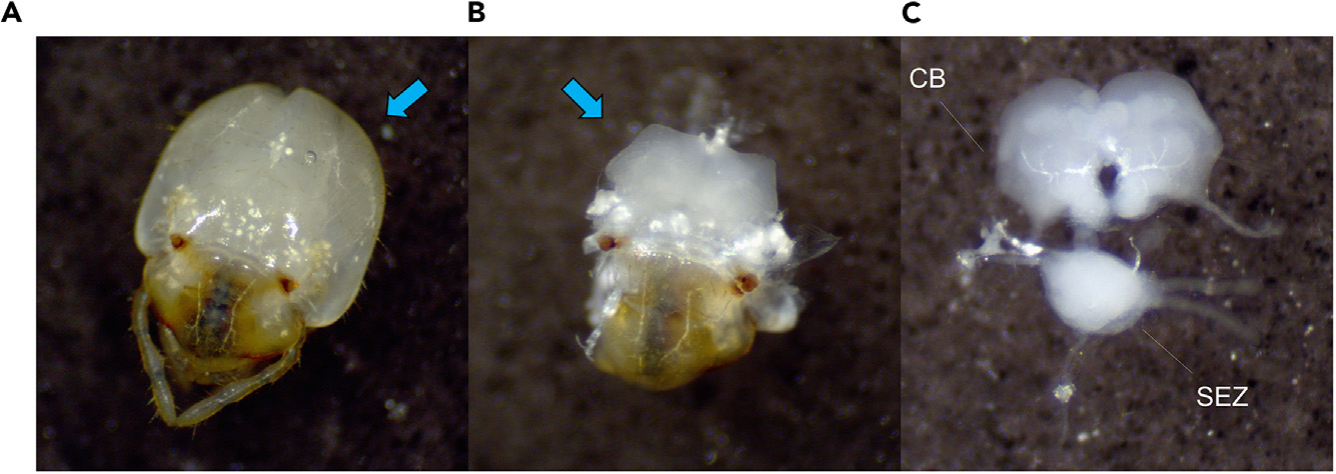
Dissection of termite brain Images were captured using an AmScope MU1803 Microscope Digital Camera. (A) Head of a termite worker. Heads should be grasped near the posterior end, indicated by the arrow, to avoid damaging the brain. (B) Partially dissected worker head. The translucent mass indicated by the arrow is the brain. Note the positions of the mandible and spots of pigmentation relative to A. (C) Fully dissected brain. The central brain (CB) and subesophageal zone (SEZ) are labeled.

***Note:*** Though dissection technique varies among individuals, it is recommended to first remove the head from the body before removing the brain. Then, while maintaining a grip on the posterior end of the head with one pair of forceps, use the other to cut the cuticle along the sides of the head. After the cuticle on top of the head is removed, the brain should be visible and the surrounding tissue, which is primarily translucent, feathery muscle, can be more easily removed.

***Note:*** Any tracheal tissue attached to the brain, which appears as solid white, fibrous strings, should be removed as long as doing so will not damage the brain.

***Note:*** The subesophageal zone (SEZ) is separate from the central brain in termites. If interested in imaging both the central brain and SEZ, exercise additional caution to keep both structures intact.

1. Anesthetize termites by placing them in a microcentrifuge tube or similar container, then submerging in ice for >2 min until termites are no longer moving.
2. Dissect brains in dissection dish containing phosphate-buffered saline (PBS) (Figure 1).

3. Using the dissecting forceps, place brains in a 2 mL microcentrifuge tube filled with PBS and kept on ice.

***Note:*** To avoid damaging the brains, do not grab them with the forceps. Instead, use the sides of the forceps to “scoop” up brains so that they can be transferred to the microcentrifuge tube.

***Note:*** If brains are kept in ice-cold PBS, it is possible to perform dissections for 3–4 h without significantly affecting results, though better results are expected the less time passes between dissection and fixation.

4. In the same microcentrifuge tube, replace PBS with 1.5 mL 2% PFA and fix for 55 min.

**CRITICAL:** Here and in all other “fix”, “wash”, or “incubate” steps, nutate the tube(s) containing the brains using an orbital shaker set to approximately 100–120 RPM.

**CRITICAL:** Here and in all other steps where the liquid contents of the tube are replaced, use a pipette to remove as much liquid as possible while taking care not to draw in any of the brains. It is more important to avoid damaging the brains than to remove all of the liquid. A 1000 μL pipette is generally sufficient, although in steps where <200 μL of liquid is used (Step 6, Step 7, etc.) a 200 μL or smaller pipette is recommended because smaller pipettes provide greater precision regarding how much liquid is removed and how quickly it is removed, thus making it easier to avoid disturbing the brains.

5. Wash brains in 10% PBST three times for 15 min each.
6. Incubate brains in 100 μL 5% normal goat serum (NGS) for 1 h.
  a. Mix 95 μL PBST with 5 μL NGS.
7. Incubate brains in 102 μL primary antibody, mouse anti-nc82, at 1:50 at 4°C, for 18–24 h.
  a. Mix 95 μL PBST with 5 μL NGS and 2 μL mouse anti-nc82.

***Note:*** If using additional primary antibodies and co-staining, they should be included in this step. Additional primary antibodies should not be derived from either mouse or goat if using the antibodies listed in this protocol and NGS.

***Note:*** Alternatives to anti-nc82, such as anti-Synapsin, are commercially available, although some adjustment of reagent concentrations may be necessary to produce satisfactory staining results.

### Day 2: Staining, continued

**Timing: 1 h**

Brains are washed in PBST to clean them of leftover reagents, then incubated in the secondary antibody, which binds to the primary antibody and possesses a fluorescent tag.

8. Wash brains in PBST three times for 15 min each.
9. Incubate brains in 100 μL secondary antibody, Alexa Fluor 546 goat anti-mouse IgG, at 1:500 at 4°C for 18–24 h.
  a. Mix 95 μL PBST with 5 μL NGS and 0.2 μL Alexa Fluor 546 goat anti-mouse IgG.

***Note:*** If using additional secondary antibodies and co-staining, they should be included in this step. Take care to ensure that fluorescence spectra do not overlap. There are many online resources that can be used to check for spectra overlap when designing experiments, for example https://www.thermofisher.com/order/fluorescence-spectraviewer/.

### Day 3: Tissue fixation, mounting, and embedding

**Timing: 7.5 h**

Brains are fixed in PFA to strengthen tissue and washed in PBST to clean them of leftover reagents, then mounted onto a coverslip, dehydrated in ethanol (xylene is soluble in ethanol but not water), cleared in xylene to increase tissue transparency, and embedded in *p*-xylene-bis-pyridinium bromide (DPX) for long-term sample preservation.

10. Wash brains in PBST three times for 15 min each.
11. Fix brains in 4% PFA for 4 h.

**Pause Point:** If time is limited, brains can be kept in PBS at 4°C following fixation, and the remainder of the protocol can be completed the next day.

**Figure 2:**
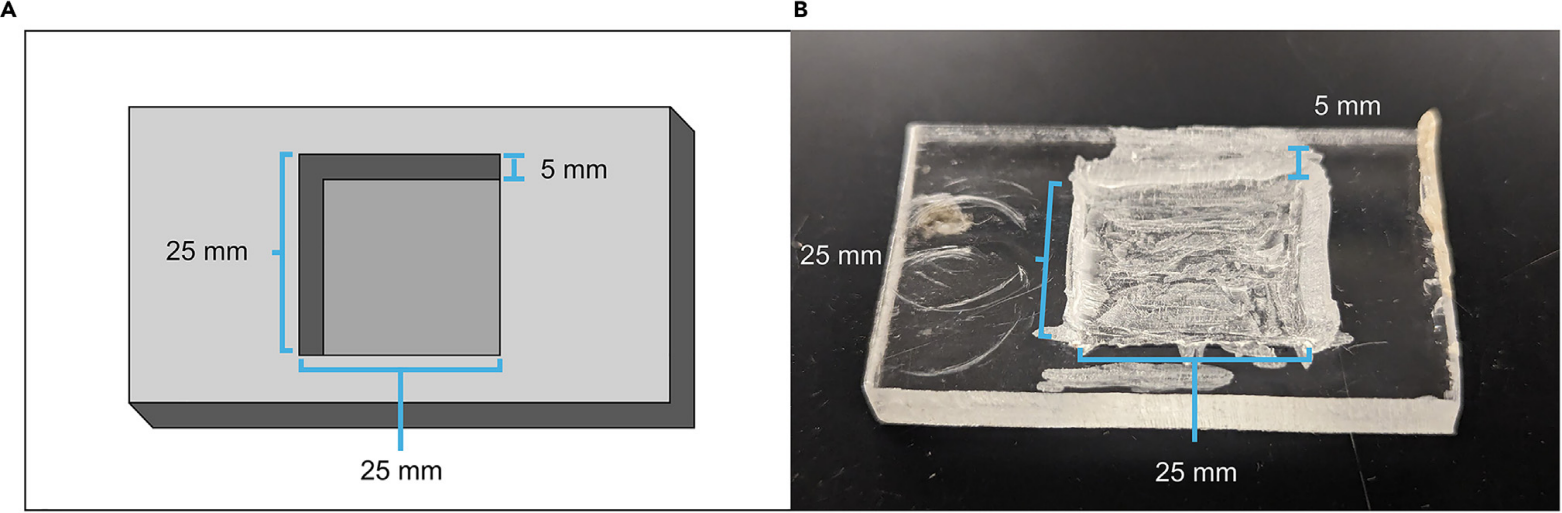
Plexiglass block used for mounting brains to coverslip When mounting brains to the coverslip, it is helpful to have some way to hold the coverslip in place. While rudimentary, we used a Plexiglass block with a shallow, coverslip-sized hole cut into it to ease the mounting process. Dimensions given are approximate. (A) Schematic drawing. (B) Photograph of block.
  b. Using a 200 μL or smaller pipette, carefully transfer each brain to the coverslip.
    ***Note:*** Arranging the brains into a grid-like pattern will make it easier to keep track of which brains have already been imaged, as well as to locate brains that need to be re-imaged. Brains should generally be oriented with either the dorsal or ventral side facing up and can be gently nudged into the desired orientation using the sides of dissecting forceps.
14. Immerse the coverslip in ultrapure water for approximately 5 s.
15. Dehydrate the coverslip in glass coverslip jars containing graded ethanol for 5 min per jar.
  a. Use the following concentrations of ethanol (diluted with ultrapure water), from beginning to end: 30%, 50%, 70%, 95%, 100%, 100%, 100%.

***Note:*** The use of three 100% ethanol jars is important to ensure that the coverslip is properly exposed to 100% ethanol. Because the coverslip is transferred directly from the 95% ethanol jar to the first 100% ethanol jar, some amount of water is inevitably transferred with it, effectively diluting the first jar to <100% ethanol. By using three 100% ethanol jars, the amount of water present in the final jar is close enough to zero to sufficiently dehydrate tissue.

12. Wash brains in PBST three times for 15 min each.
  a. Simultaneously, prepare a poly-L-lysine coated coverslip.
    ***Optional:*** Using dissecting forceps, break off a small section of one of the corners of the coverslip to make it easier to keep track of its orientation.
    i. Fully immerse the coverslip in the glass coverslip jar containing poly-L-lysine solution for approximately 5 s.
    ii. Gently shake the coverslip to remove excess solution for approximately 10–30 s.
    iii. Place coverslip on a coverslip rack to dry for 15 min.
    iv. Repeat twice, for a total of three immerse/dry cycles.
13. Mount brains onto the coverslip.
  a. Immerse the coverslip in PBS so that it lies flat.
    ***Note:*** It is important that the coverslip be held steady and fully immersed in PBS while mounting brains. Janelia Research Campus provides a design for a mounting dish (https://www.janelia.org/open-science/drosophila-mounting-t-dish) that can be manufactured if one has access to the necessary tools or used as a basis for more rudimentary solutions. For example, cutting a shallow, coverslip-sized well into a block of Plexiglass or a similar material creates a simple mounting dish (Figure 2).

16. Within a fume hood, clear the coverslip in glass coverslip jars containing xylene for 5 min per jar.
  a. Use three jars, each containing 100% xylene.

***Note:*** The same rationale for using three jars applies here as with the 100% ethanol jars. Because the coverslip is transferred directly from the final 100% ethanol jar to the first 100% xylene jar, some amount of ethanol will be transferred and dilute the xylene. Using three jars maximizes the xylene concentration in the final jar and ensures that tissue is sufficiently cleared.

**Figure 3:**
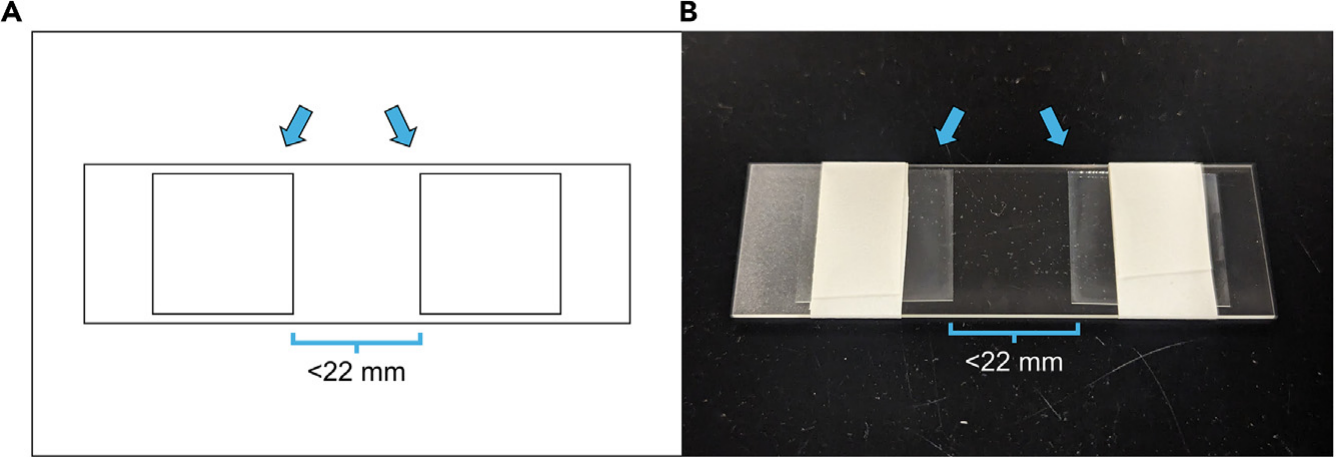
Example slide with makeshift spacers Spare coverslips are adhered to the slide just under one coverslip’s width apart from each other using masking tape. During DPX embedding, these coverslips act as spacers that prevent the brains from being crushed between the slide and the coverslip they are mounted on. Arrows highlight coverslips. (A) Schematic drawing. (B) Photograph of slide.
18. Allow slide to dry in darkness at room temperature within a fume hood for at least 2 days before imaging.
  a. Use the lid of a cardboard microtube box (or similar) to cover the slide while drying.

***Note:*** After drying, keep slides in a box to prevent light exposure. Slides can be imaged for up to 4–6 months after drying.

17. Within a fume hood, pipette 3–4 drops of DPX onto the side of the coverslip with the brains mounted, then place the coverslip brain side down onto a slide prepared with spacers.
  **CRITICAL:** Xylene evaporates quickly and it is important that the brains do not dry out before DPX application. For this reason, DPX should be applied as soon as possible after removing the coverslip from the final xylene jar.
  ***Note:*** Spare coverslips can be used as spacers by placing two just under a single coverslip’s width apart from each other, then adhering them to a slide with masking tape (Figure 3). The coverslips listed in the key resources table are approximately 130–160 μm thick, while dehydrated *Reticulitermes flavipes* brains are approximately 80–100 μm thick. If insects with significantly larger brains are examined, care should be taken when selecting spacers to avoid crushing the brains.
  a. Gently press down on the coverslip using a spare slide to ensure that coverslip adheres to the slide.

19. Image brains on slides using confocal microscopy.

***Note:*** Microscopes vary regarding the ideal image capture settings, although we recommend capturing images at 1 μm intervals if observing the whole brain, while a pixel resolution of 1024 × 1024 is generally adequate. If oriented dorsal or ventral side up, *R. flavipes* brains are approximately 500–600 μm wide, 300–400 μm long, and 100–120 μm thick, so the objective used should be chosen accordingly. A line averaging setting of 2 (if available on the microscope used) is helpful in reducing noise.

## Expected outcomes

**Figure 4:**
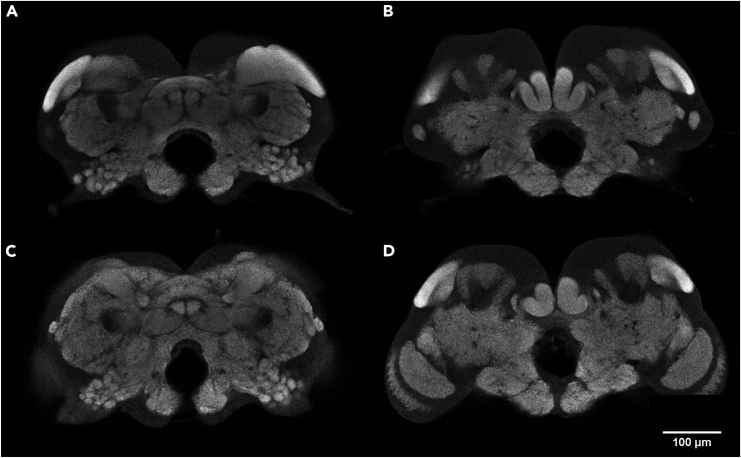
Representative images of immunostained *R. flavipes* brains Images were captured using a Leica SP8 DLS laser scanning confocal microscope with a 10× dry objective (HC PL APO 10×/0.40) at 1024 × 1024 pixel resolution and 0.62 × 0.62 × 1 μm voxel size, scale bar: 100 μm. (A) Worker brain at depth of approximately 40 μm. (B) The same worker brain at depth of approximately 60 μm. (C) Alate brain at depth of approximately 40 μm. (D) The same alate brain at depth of approximately 70 μm.

Following the protocol as described here will produce immunostained termite brains that can then be imaged via confocal microscopy (Figure 4; Methods videos S1 and S2). Ultimately, how images produced through this protocol are used is up to the goals of the researcher. Images produced using nc82 as the only primary antibody, for example, can be used in volumetric analyses of gross brain anatomy or in the generation of standard brains, both of which were employed in our comparison of brain allometry between termite castes.^1^ If additional antibodies are employed, the expression of specific proteins can be localized, although a current lack of information regarding primary antibodies known to be compatible with termite brains means that attempting such analyses will require preliminary trial and error runs.

Methods video S1. Representative confocal stack of *R. flavipes* worker brain, related to expected outcomes Images captured using a Leica SP8 DLS laser scanning confocal microscope with a 10× dry objective (HC PL APO 10×/0.40) at 1024 × 1024 pixel resolution and 0.62 × 0.62 × 1 μm voxel size.

Methods video S2. Representative confocal stack of *R. flavipes* alate brain, related to expected outcomes Images captured using a Leica SP8 DLS laser scanning confocal microscope with a 10× dry objective (HC PL APO 10×/0.40) at 1024 × 1024 pixel resolution and 0.62 × 0.62 × 1 μm voxel size.

## Limitations

In general, the protocol described here is fairly lenient in that, once the baseline conditions to produce images are determined, minor changes in the timing of steps or exact quantities of reagents should not significantly impact the results. In addition, given that the protocol is heavily derived from one used in *Drosophila*,^3^ it should be applicable to other insects following additional adjustment. For this reason, however, it may take some amount of trial and error to produce images of the desired quality. This protocol is also relatively fast compared to similar protocols that, for example, utilize incubation periods of 48 h rather than the 18–24 h recommended here, which may impact staining quality.^10^^,^^11^^,^^12^

## Troubleshooting

### Problem 1

General difficulties associated with performing microdissections.

### Potential solution

The dissection step (Step 2) is the most difficult part of the protocol, but with practice should become easier to perform. Ultimately the goal is to remove an insect’s brain from its head, but the specific steps taken to achieve this are relatively unimportant as long as the brain is removed and intact. At a baseline level, however, it is important that the dissector’s hands remain as steady as possible, or else it will be nearly impossible to perform efficient and accurate microdissections. Resting one’s arms on the table that the microscope is placed on, so that the entire arm from elbow to wrist is in contact with the table, helps significantly in reducing shakiness of the hands. It is also important to control one’s breathing, particularly during very delicate steps. Taking long, deep breaths minimizes movement of the body while also potentially helping to calm one’s nerves.

### Problem 2

Damaged brains.

### Potential solution

Damage to brains can occur at any point in the protocol but is particularly likely during dissection (Step 2) and mounting (Step 13). When manipulating brains with the dissecting forceps, such as when moving them into place on the coverslip, never allow the tips of the forceps to contact the brains and do not directly grasp the brains with the forceps. Instead, use the rounded edges of the forceps to gently nudge brains into the desired position, or lift them up from below.

### Problem 3

Brains adhere to sides of microcentrifuge tube or pipette tip.

### Potential solution

If brains adhere to the microcentrifuge tube, they can usually be gently pushed off using the blunt sides of dissecting forceps, although doing so always risks causing damage to brain tissue. Ideally, preventative measures should be taken to minimize adhesion of brains to other surfaces. If tissue adhesion is a reoccurring issue, it is recommended to try using a different brand/type of microcentrifuge tube. Eppendorf Protein LoBind Tubes show low adhesion and are recommended for this reason, although other types of tubes may also show low adhesion. Adhesion can be tested by placing a single brain into a PBS-filled tube and gently shaking it so that the brain brushes against the walls of the tube. Adhesion to the pipette tip is more difficult to control and care should be taken when replacing the liquid in the tube not to draw in any brains. When transferring brains to the coverslip, cutting off the end of the pipette tip (blunting it) can lower the risk of damaging tissue by creating a large opening for brains to pass through. Brains will generally show reduced adhesion to plastic surfaces as the protocol progresses due to Triton exposure.

### Problem 4

Brains do not adhere to coverslip.

### Potential solution

One possibility is that the poly-L-lysine solution being used has expired and needs to be replaced. Ideally, the solution will remain usable for up to 12 months, although this length of time may be altered by storage conditions. The molecular weight of the poly-L-lysine used can also influence adhesion. It is recommended to use poly-L-lysine with a molecular weight >300,000 for this reason.

### Problem 5

**Figure 5:**
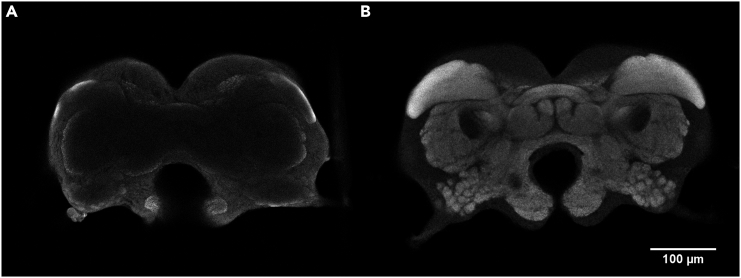
Influence of PBST concentration on imaging results In some cases, imaging results may show a concentration of antibody signal around the periphery of the brain, with low penetration. One potential solution is to increase the concentration of Triton X-100 used in PBST washes. Images were captured using a Leica SP8 DLS laser scanning confocal microscope with a 10× dry objective (HC PL APO 10×/0.40) at 1024 × 1024 pixel resolution and 0.62 × 0.62 × 1 μm voxel size, scale bar: 100 μm. Both images were captured at approximately the same depth. (A) *R. flavipes* worker brain treated with 0.3% PBST. (B) *R. flavipes* worker brain treated with 10% PBST; remainder of protocol identical to A.

Antibody signal appears to congregate around the periphery of the brain with low penetration (Figure 5).

### Potential solution

Antibody penetration can be increased by increasing the concentration of Triton X-100 used in the PBST recipe. The concentration given throughout this protocol, 10%, is significantly higher than that used in similar protocols in other insects,^3^^,^^10^^,^^11^^,^^13^ including other termite species,^14^ which generally use Triton X-100 concentrations of 0.3–1.0%. Thus, it is worth experimenting with different PBST concentrations to observe their impact on staining results. In our case, an initial concentration of 0.3% Triton X-100 resulted in low antibody penetration. By increasing the Triton X-100 concentration to 0.5%, then by 0.5% steps, we eventually arrived at the 10% Triton X-100 concentration used here, which provided satisfactory levels of antibody penetration. Given the relatively short incubation period (18–24 h) recommended here, increasing this period in increments of 24 h may also improve antibody penetration, although we have not directly tested this.

## Resource availability

### Lead contact

Further information and requests for resources and reagents should be directed to and will be fulfilled by the lead contact, Xuguo Zhou (xgzhou@illinois.edu).

### Technical contact

Technical questions on executing this protocol should be directed to and will be answered by the technical contact, Austin Merchant (ajme232@g.uky.edu).

### Materials availability

This study did not generate new unique reagents.

### Data and code availability

The confocal data generated during this study is available at Dryad: https://doi.org/10.5061/dryad.m37pvmd9x.

## Acknowledgments

This work was supported by the 10.13039/100007917US Department of Agriculture, 10.13039/100005825National Institute of Food and Agriculture, Agriculture and Food Research Initiative (USDA-NIFA-AFRI) Predoctoral Fellowship (award no. 2021-67034-34975) to A.M., the William L. and Ruth D. Nutting Research Grant awarded by the International Union for the Study of Social Insects, North America Section to A.M., and the 10.13039/100005825USDA-NIFA Hatch projects (KY008071 and KY008090) to X.Z.

## Author contributions

A.M. and X.Z. designed the study; A.M. conceived the experiments, analyzed the data, and drafted the manuscript; and X.Z. revised and finalized the manuscript. All authors read and approved the final version of the manuscript.

## Declaration of interests

The authors declare no competing interests.

## References

[1] Merchant A., Zhou X. (2024). Caste-biased patterns of brain investment in the subterranean termite Reticulitermes flavipes. iScience.

[2] Kinoshita M., Homberg U., Shigeno S., Murakami Y., Nomura T. (2017). Brain Evolution by Design: From Neural Origin to Cognitive Architecture.

[3] Zhou C., Pan Y., Robinett C.C., Meissner G.W., Baker B.S. (2014). Central brain neurons expressing doublesex regulate female receptivity in Drosophila. Neuron.

[4] Jefferis G.S.X.E., Potter C.J., Chan A.M., Marin E.C., Rohlfing T., Maurer C.R., Luo L. (2007). Comprehensive maps of Drosophila higher olfactory centers: Spatially segregated fruit and pheromone representation. Cell.

[5] Rein K., Zöckler M., Mader M.T., Grübel C., Heisenberg M. (2002). The Drosophila standard brain. Curr. Biol..

[6] Im K., Mareninov S., Diaz M.F.P., Yong W.H., Yong W.H. (2019). Biobanking: Methods and Protocols.

[7] Govorushko S. (2019). Economic and ecological importance of termites: A global review. Entomol. Sci..

[8] Wagh D.A., Rasse T.M., Asan E., Hofbauer A., Schwenkert I., Dürrbeck H., Buchner S., Dabauvalle M.-C., Schmidt M., Qin G. (2006). Bruchpilot, a protein with homology to ELKS/CAST, is required for structural integrity and function of synaptic active zones in Drosophila. Neuron.

[9] Meissner, G. (2016). FlyLight Recipe - PLL Solution. https://www.janelia.org/sites/default/files/FL%20Recipe%20-%20Poly-L-Lysine%20.pdf.

[10] Lucas C., Kornfein R., Chakaborty-Chatterjee M., Schonfeld J., Geva N., Sokolowski M.B., Ayali A. (2010). The locust foraging gene. Arch. Insect Biochem. Physiol..

[11] Wu J.S., Luo L. (2006). A protocol for dissecting Drosophila melanogaster brains for live imaging or immunostaining. Nat. Protoc..

[12] Kohl J., Ng J., Cachero S., Ciabatti E., Dolan M.-J., Sutcliffe B., Tozer A., Ruehle S., Krueger D., Frechter S. (2014). Ultrafast tissue staining with chemical tags. Proc. Natl. Acad. Sci. USA.

[13] Li J., Merchant A., Zhou S., Wang T., Zhou X., Zhou C. (2022). Neuroanatomical basis of sexual dimorphism in the mosquito brain. iScience.

[14] Valadares L., da Silva I.B., Costa-Leonardo A.M., Sandoz J.-C. (2023). Differentiation of workers into soldiers is associated with a size reduction of higher-order brain centers in the neotropical termite Procornitermes araujoi. Sci. Rep..

